# Birth mass is the key to understanding the negative correlation between lifespan and body size in dogs

**DOI:** 10.18632/aging.101081

**Published:** 2016-12-08

**Authors:** Rong Fan, Gayla Olbricht, Xavior Baker, Chen Hou

**Affiliations:** ^1^ Biology Department, Missouri University of Science and Technology, Rolla, MO 65409, USA; ^2^ Second Hospital Affiliated to Heilongjiang University of Chinese Medicine, Harbin, 150001, China; ^3^ Mathematics and Statistics Department, Missouri University of Science and Technology, Rolla, MO 65409, USA

**Keywords:** metabolic energy, health maintenance, growth, tradeoff, scaling law

## Abstract

Larger dog breeds live shorter than the smaller ones, opposite of the mass-lifespan relationship observed across mammalian species. Here we use data from 90 dog breeds and a theoretical model based on the first principles of energy conservation and life history tradeoffs to explain the negative correlation between longevity and body size in dogs. We found that the birth/adult mass ratio of dogs scales negatively with adult size, which is different than the weak interspecific scaling in mammals. Using the model, we show that this ratio, as an index of energy required for growth, is the key to understanding why the lifespan of dogs scales negatively with body size. The model also predicts that the difference in mass-specific lifetime metabolic energy usage between dog breeds is proportional to the difference in birth/adult mass ratio. Empirical data on lifespan, body mass, and metabolic scaling law of dogs strongly supports this prediction.

## INTRODUCTION

The rate of living theory, one of the oldest theories of aging, suggests that the mass-specific lifetime energy expenditure of organisms is independent on body mass [1, 2]. Two interspecific scaling laws of mammals provide strong support to this theory. The mass-specific field metabolic rate, which is equivalent to the average rate of daily energy expenditure (*DEE*), generally scales with body mass to a power around −0.25 across mammalian species with the body mass ranging from 7 to 100,000 grams [3, 4], whereas the scaling power of lifespan is roughly +0.25 [5] or slightly lower (+0.21) [6]. Thus, larger mammalian species have lower mass-specific daily energy expenditure rate but longer lifespan than smaller ones. Consequently, with a few exceptions, the product of these two traits, which gives the lifetime energy usage per body mass, is approximately a constant across species.

**Figure 1:**
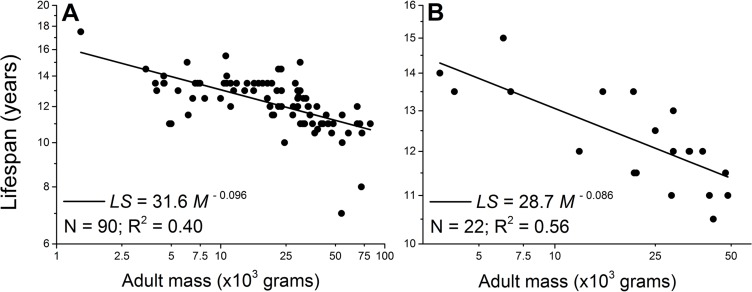
Lifespan negatively scales with adult body mass in male (A) and female dogs (B) Each point represents one breed. The scaling powers were obtained by regressing the logarithmically transformed data.

However, intra-specific scaling laws of a broad range of dog breeds challenge the theory. Mass-specifically, the dog's metabolic scaling power is −0.31 [7, 8]. According to the rate of living theory, the lifespan of dogs would scale with body mass to a power around 0.30, i.e., larger dogs would live longer. But an opposite trend has been well-documented [7, 9-13]. For example, Comfort [14] found that in four breeds of dogs, the scaling power of maximum lifespan is −0.15, and data collected in this study show a −0.096 scaling power for the average lifespan across 90 breeds of dogs (Figure 1 and Supplementary data).

The negative correlation between longevity and body mass in dogs has been a long-standing question in the study of aging [7, 9, 12]. Similar negative correlations have been noticed in other species, such as rodents [15] and humans [16]. But most research on this topic remains either descriptive, such as a few statistical analysis [7, 10, 13, 15, 17], or qualitative, such as the thermoregulation hypothesis [9], which postulates that because of the high surface-to-volume ratio, small dogs spend more energy to generate heat by decoupling proton transport from ATP generation, and therefore have lower production of deleterious oxygen free radicals. Still largely missing is the answer to the key question: why do interspecifically smaller mammals live shorter, while intra-specifically, smaller breeds or strains live longer than larger ones? None of the previous studies, descriptive or mechanistic, offered a general theory that is able to reconcile these opposite trends within the same framework, and make quantitative predictions.

Here, we apply a theoretical model based on the first principle of energy conservation to reveal the energetic mechanism underlying this paradox. The key idea of the model lies in the tradeoff between the energy allocations to biosynthesis during growth and health maintenance. The quantitative predictions of the model are well-supported by empirical data on body mass, metabolic rate, and lifespan from a broad spectrum of wild animals and more than 200 studies on laboratory rodents that are under food restriction or genetically manipulated [18, 19]. Applying this model to dogs, we show that when searching for the explanations for the negative correlation between longevity and adult body mass of dogs, all the previous studies have ignored the ratio of birth mass and adult mass, which is the key to understanding this issue. A lower ratio indicates that animals spend relatively more energy on growth to reach adult size, and therefore will have relatively less energy for health maintenance efforts, such as scavenging free radicals and repairing oxidative cellular damage. Consequently, the breed with a lower birth/adult mass ratio will have a shorter lifespan.

The tradeoff between growth and longevity has been investigated in many intra-specific studies. Rapid growth promotes a series of oxidative cellular damage, such as increased phospholipid peroxidation [20], increased protein carbonyl content [21], decreased antioxidant defenses in red blood cells [22], and elevated free radical processes [23]. The growth-induced cellular damage accumulates during development, and has long-term adverse effects on animals’ health maintenance and longevity, even in species, whose developmental stage is much shorter than lifespan, such as rodents and humans [24-27]. Several theoretical efforts have been made to understand the negative correlation between growth and longevity (e.g., [27-31]). Most of the previous works employ evolutionary approaches, and are more or less qualitative. The model we present here does not consider reproductive success or the external mortality rate (though they can be potentially included in this model, see [32]). Our model focuses on the physiological basis of growth and longevity. Using only one free-floating parameter, which can be verified independently, the model accurately predicts the relationship between birth mass, adult mass, metabolic rate, and lifespan of dogs. More importantly, the model reveals a general theory, and suggests that the conventional interspecific rate of living theory and the intra-specific negative correlations between lifespan and body size observed in dogs and rodents under food restriction are all special cases, which can be explained by one general equation simultaneously.

### The theoretical model

The model is based on three assumptions [18, 19, 33, 34].

Assumption 1: Oxidative metabolism produces free radicals such as reactive oxygen species (ROS), which cause damage to macromolecules [35, 36]. We assume that the rate of damage production, *H*, is proportional to field metabolic rate (equivalent to daily energy expenditure), *B*, with a coefficient *δ*, i.e., *H* = *δB*. The proportionality may not always hold when comparison is made across taxa, e.g., mammal versus bird, or under short-term stressful conditions, such as heavy exercises and cold exposure [35]. But averaging over lifetime of dogs living in normal domestic environments, this assumption is generally valid [18, 19, 37-39]. Note: here *H* refers to the raw damage including the raw ROS production, before scavenging and repair are taken into consideration. The net damage (raw minus repair) may or may not be positively correlated to metabolic rate (see below).

Assumption 2: Organisms have evolved mechanisms to scavenge radicals and repair cellular damage, which cost metabolic energy. We assume that the rate of scavenging/repair, *R*, is proportional to the rate of energy available for health maintenance, *B*_maint_, with a coefficient *η*, i.e., *R* = *ηB*_maint_. Numerous energy budget models and empirical data (e.g., [40-42]) suggest that the resting metabolic energy (*B*_rest_) is partitioned between the energy for maintenance (*B*_maint_) and the energy required for biosynthesizing new tissues during growth (*B*_syn_), i.e., *B*_maint_ = *B*_rest_ − *B*_syn_. This partition lays the foundation of the tradeoff between biosynthesis and maintenance. For free-living animals, the ratio of resting metabolic rate (*B*_rest_) and field metabolic rate is roughly a constant, i.e., *B* = *f* × *B*_rest_, where *f* is about 2 to 3 and independent of body mass [3, 40, 43]. Combining Assumptions 1 and 2, we have the net damage *H* − *R* = *δfB*_rest_ - *η*(*B*_rest_ − *B*_syn_) = (*δf* − *η*)*B*_rest_ + *ηB*_syn_. If a large amount of metabolic energy is allocated to biosynthesis (*B*_syn_) during growth, then the maintenance effort (*B*_maint_ = *B*_rest_ -*B*_syn_) is small, and the net damage is high, as seen in the equation above (the term of +*ηB*_syn_). Thus, while we assume the raw damage and ROS to be proportional to metabolic rate *B*, we do not make such an assumption for the net damage. Our assumption is yet to be tested. Unfortunately, it is difficult to determine whether the empirically measured damage or ROS is raw or net. For example, Salin et al [44] employed a newly developed technique to measure the production of H_2_O_2_ (a major ROS) in fish mitochondria *in vivo*, and found that individual fish with higher metabolic rates have lower levels of ROS. However, as suggested by Salin et al [44], although the negative correlation may reflect the effect of mitochondrial proton-leak, which may cause low ROS production when metabolic rate is high, “it is feasible that individuals with a lower H_2_O_2_ level may have allocated more resources towards antioxidant defences”[44]. We call for future studies to consider the tradeoff between biosynthesis and maintenance when investigating the correlation between metabolic rate and damage/ROS production [19, 45].

The net damage accumulates as an integral of time, $\int {}_{0}^{t}(H\;-\;R)d\tau =\;\int {}_{0}^{t}[(\delta f\;-\;\eta )B_{rest}\;+\;\eta B_{syn}]d\tau$. Resting metabolic rate *B*_rest_ scales with body mass as *B*_rest_ = *B*_0_*m*(*t*)^α^, where *m*(*t*) is body mass as a function of age *t*, *B*_0_ is a normalization coefficient, and *α* is the scaling power [41, 42]. The rate of energy allocated to biosynthesizing new biomass is *B*_syn_ = *E*_m_*dm/dt*, where *dm*/*dt* is growth rate, and *E*_m_ is the energy required to synthesize one unit of bio-tissue, such as the energy for assembling macromolecules from monomers [40]. Here, synthesis only includes addition of tissues, and excludes biomolecules for replacing damaged tissues, the energy for which is included in the maintenance term *B*_maint_ [42]. We now define the ratio of damage repair rate and gross damage generation rate, *ε*=*η*/(*fδ*), as the protective efficiency. A higher *ε* indicates a higher capacity of damage repairing. Using these relationships, the integral gives the normalized net mass-specific cellular damage as a function of age, *t*,

$$\begin{array}{l} D(t)\;=\;1\;/\;m(t)\;\times \;{\int^{\text{​}}}_{0}^{t}[(1\;-\;\varepsilon )B_{rest}\;+\;\;\varepsilon B_{syn}]d\tau \\ \;\;\;\;\;\;\;\;\;\;\;\approx (1\;-\;\varepsilon )B_{0}m{\left(1\right)}^{\alpha -1\;}\;\times \;\;t\;\;+\;\;\varepsilon \;E_{m}[m(t)\;-\;m_{0}]\;/\;m(t) \end{array}$$ (1)

where *m*_0_ is birth mass at *t* = 0. The first term in Eq. 1 is approximate, i.e., $\int {}_{0}^{t}\;m{\left(\tau \right)}^{\alpha }d\tau \;\approx \;m{\left(t\right)}^{\alpha }\;\times t$. The exact analytic result of this integration is available in [18] (Eq. 5 in this reference). The approximation is accurate for an age *t* close to the lifespan, i.e., much larger than the age at which the adult mass is reached. The second term in Eq. 1 is estimated as $\int {}_{0}^{t}\;B_{syn}d\tau \;=\;E_{m}\int {}_{0}^{t}\;dm\;/\;|d\tau \;\times d\tau =\;E_{m}\Delta |m{|}_{0}^{t}$, and $\;E_{m}\Delta_{m}{|}_{0}^{t}\;=\;E_{m}[m(t)\;-\;m_{0}]$ expresses the net energy allocated to biosynthesis from birth to age *t*. The detailed calculation of the integral is available in [18] and [33].

**Figure 2:**
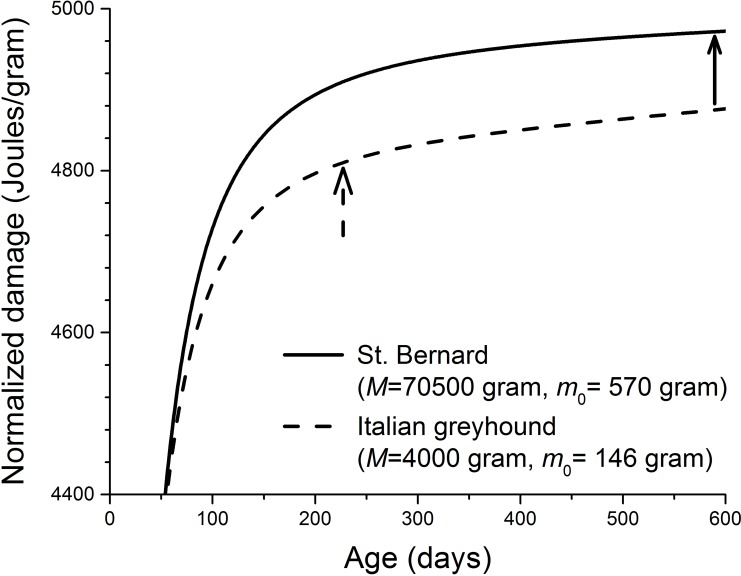
Calculated cellular damage increases as a function of age in two dog breeds (physiological parameters in Eq. 1 required to produce this figure: *B*_0_ = 3158 Joules/(day.gram^0.69^) [7, 8], *E*_m_ = 5000 Joules/gram, and *ε* = 0.999) [34, 40, 53]. The solid and dashed arrows indicate the ages at which ∼ 90% of the adult masses are reached in two dog breeds. These ages were estimated from the growth equation, *E*_m_*dm / dt* = *B*_rest_ − *B*_maint_ and the birth and adult masses of both dog breeds.

Equation 1 estimates a theoretical profile of the damage accumulation during ontogeny. The growth curve *m*(*t*) can be determined by the equation *E*_m_*dm* | dt = *B*_rest_ - *B*_maint_, once four parameters are empirically given, namely, birth mass *m*_0_, adult mass *M*, energy required to synthesize one unit of biomass *E*_m_, and metabolic normalization constant *B*_0_. The theoretical predictions of growth curves were generally supported by empirical data from a variety of species [42]. In Figure 2, we use Eq. 1 and the physiological values of *m*_0_, *M*, *E*_m_, and *B*_0_ of two breeds of dogs (small and large) to show that damage increases as a function of age *t*. Figure 2 shows that cellular damage level increases fast during development, and slows down after adult size is reached. In [34], we gave detailed reasons for the shape of the damage curve. Here we explain it briefly as the following: In Eq. 1, two terms contribute to the cellular damage, the metabolic term (1 − *ε*)*B*_0_*m*(*t*)^*α*−1^×*t*, and the biosynthetic term *εE*_m_[*m*(*t*) − *m*_0_]/*m*(*t*). Based on the first principle of biochemistry and fitting of empirical data from rodents, the protective efficiency *ε* has been estimated to be very high, and close 0.99 ([18, 33]. Thus, the coefficient of the metabolic term (1 − *ε*) is much smaller than that of the biosynthetic term *ε*. This suggests that if the energy for repair is unlimited, the highly efficient repairing mechanism will repair most of the damage, so that damage accumulates at a low rate that is proportional to (1 − *ε*). However, during growth, biosynthesis costs a considerable amount of energy that could be allocated to repair otherwise, so that damage accumulates at a fast rate (*ε*) despite the highly efficient repairing mechanism, and biosynthesis (the second term in Eq. 1) is the major contributor to damage during growth. Note: the damage curves in Figure 2 are predicted based on the theoretical estimates of growth curves. If the empirical growth curves are used, the damage curves will change accordingly, but the qualitative nature of the shape, i.e., increasing fast during growth and slow during adulthood, will not change. Unfortunately, very limited empirical works have been conducted to test the predicted shape of damage profile over ontogeny. One available example is that the lipid peroxidation level in mice brain increases considerably faster during development than during adulthood [23] (and see analysis in [19]).

The cellular damage in our model is general. It includes the oxidative assaults on lipid, protein, and DNA, which are predicted to increase rapidly during growth and then slow down once adulthood is reached (Figure 2). The existing evidence to test this prediction is limited. Here we suggest that telomere length can be a good candidate for biomarker of cellular damage to test this prediction directly. Telomere length declines with DNA replication in cells that lack enzyme telomerase. One important cause of the telomere shortening, besides the “end-replication problem”, is oxidative stress, which causes DNA single-strand-breaks (SSB) [46-49]. The repair of SSB in telomere is imperfect, and the unrepaired SSB are lost during cell replication, and that results in the shortening of telomere [48, 50]. It is possible that during growth, due to the insufficient energy and resource, the SSB repair is inefficient and the length of telomere declines rapidly, and the rate of decline slows as animals mature. It has been found that across 15 dog breeds, the telomere length in blood mononuclear cells is a strong predictor of lifespan [51]. However, the existing data on the rate of telomere loss have low temporal resolution, and in many cases are only available in adult animals. Thus, we call for future studies to assay the profiles of telomere length over ontogeny.

Assumption 3: We assume that animals die when the mass-specific cellular damage level *D*(*t*) reaches a threshold *C*, i.e., *D*(*t* = *LS*) = *C*, where *LS* is lifespan, and *C* is a constant for all dog breeds. A similar assumption of the damage threshold for loss of functions or mortality has been made by Sohal and colleagues [52]. We will show below that it is unnecessary to know the exact value of the threshold to make quantitative predictions.

Now we compare two breeds of dogs, denoted by *i* and *j*. Assumption 3 suggests that when lifespan is reached, these two breeds of dogs will have the same damage level, i.e., *D*(*LS*_i_) = *D*(*LS*_j_) = *C*. Substituting Eq. 1 into this relationship, we have

$$\left(1-\varepsilon )B_{0}M_{i}^{\alpha -1}LS_{i}+\varepsilon E_{m}(1-\mu_{i})=(1-\varepsilon )B_{0}M_{j}^{\alpha -1}LS_{j}+\varepsilon E_{m}(1-\mu_{j}\right)$$

where *μ* = *m*_0_/*M* is the ratio of birth and adult mass. A higher *μ* indicates that less energy is allocated to biosynthesis, and therefore less damage is accumulated. We can rewrite this equation as

$$B_{0}M_{i}^{\alpha -1}LS_{i}-B_{0}M_{j}^{\alpha -1}LS_{j}=\frac{\varepsilon E_{m}}{1-\varepsilon }(\mu_{i}-\mu_{j})$$ (2)

Equation 2 is our main theoretical result. *B*_0_*M*^*α*−1^*LS* on the left hand side is nothing but mass-specific lifetime energy usage, which we will denote as *LE* for convenience. Thus, Eq. 2 makes a simple prediction: the difference in *LE* (Δ*LE* = *LE_i_* − *LE_j_*) between breeds is proportional to the difference in birth/adult mass ratio *μ* (Δ*μ* = *μ_i_* − *μ_j_*) with a constant, *E*_m_*ε*/(1 − *ε*).

It is straightforward to test this prediction. We need to emphasize that Eq.2 has only one free floating parameter—the species-specific protective efficiency *ε*. The normalization metabolic coefficient *B*_0_ of dogs was measured as 3158 Joules/(day.gram^0.69^) [7, 8], and the energy for synthesizing one unit of bio-tissue *E*_m_ is a constant within a species, averaging around 5000 Joules/gram in dogs and other mammals [34, 40, 53]. Previous studies have collected data on average adult mass and average lifespan of dogs, but as far as we know, data on birth mass is not available in any existing dataset. Fortunately, numerous dog owners have recorded the birth masses of a broad range of breeds. Thus, following the approach taken by many researchers (e.g., [9, 10]), who collected data on from web-based resources primarily generated by breeders, we were able to obtain data on 90 breeds of male dogs and 22 breeds of females. When multiple sources give different values for a certain breed, we took the average value. The data are available in Supplementary Material.

## RESULTS

### Birth/adult mass ratio and scaling law of lifespan of dogs

**Figure 3:**
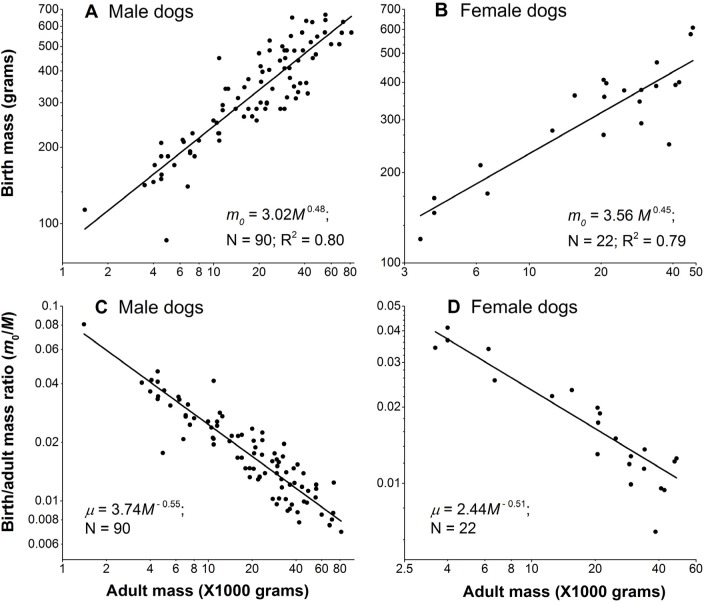
Birth mass is not proportional to adult mass in dogs (**A**) and (**B**): Birth mass scales with adult mass sub-linearly in male and female dogs, respectively. (**C**) and (**D**): Birth/adult mass ratio negatively scale with adult mass in male and female dogs, respectively. Each point represents one breed. In each gender, the sum of the absolute values of the scaling powers of the birth mass and the ratio, in principle, should be equal to 1. They are close, but not exactly equal to 1, because dividing the birth mass by the adult mass M in the ratio, μ = m_0_/M, introduces noise from M. Nonetheless, as Figure 3 shows, the noise is negligible, as the sums of the powers are 0.48+0.55 = 1.03 in males and 0.45+0.51 = 0.96 in female dogs.

Figure 3A and 3B show that the birth mass of dog scales sub-linearly with the adult mass. Consequently, the birth/adult mass ratio *μ* scales with the adult mass to powers of −0.55 and −0.51 for male and females, respectively (Figure 3C and 3D), indicating that larger dogs have relatively smaller birth mass. To obtain the scaling powers in these panels, we first logarithmically transformed the data, and then performed linear regression.

Our model suggests that the non-zero scaling power of *μ* gives rise to the negative correlation between lifespan and adult mass in dogs. Using Assumption 3 and Eq. 1, we estimate the mass-specific cellular damage level in an organism when the lifespan is reached: *D*(*LS*) = (1 − *ε*)*B*_0_*M*^*α*−1^*LS* + *εE*_m_(1 − *ε*) = *C*, where *C*, *B*_0_, *ε*, and *E*_m_ are constants. It is straightforward to express lifespan from this equation as

$$LS\propto (C-\varepsilon E_{m})M^{1-\alpha }+\varepsilon E_{m}\mu M^{1-\alpha }$$ (3).

If *μ* is a constant or weakly scales with body mass, then we have *LS* ∝ *M*^1−*α*^, which is exactly what the rate of living theory predicts: when the metabolic scaling power *α* = 0.75, the lifespan scaling power is around 0.25, and is what has been observed interspecifically. However, in dogs *μ* scales with body mass as *μ* = 3.74*M*^−0.55^, using male as an example. Using *α* = 0.69 for the metabolic scaling of dogs [7, 8], Eq. 3 becomes *LS* ∝ (*C* − *εE*_m_)*M*^0.31^ + 3.74*εE*_m_*M*^−0.24^, indicating that the scaling power of lifespan lies between 0.31 and −0.24, depending on the coefficients of these two terms, (*C* − *εE*_m_) and 3.74*εE*_m_, and the range of *M*. Unfortunately, no data is available to accurately estimate *C* and *ε* in dogs. But the theoretical calculation for general mammals suggests that *C* is slightly larger than *E*_m_, and *ε* is around 0.99, close to 1 [19, 33]. If the same is true for dogs, then the first coefficient (*C* − *εE*_m_) is much smaller than the second coefficient 3.74*εE*_m_. Considering that adult mass *M* varies between 1000 to 90,000 grams, the scaling power of lifespan predicted by Eq. 3 will lean towards a negative value, as what has been observed (Figure 1).

### Mass-specific lifetime energy usage is proportional to birth/adult mass ratio

**Figure 4:**
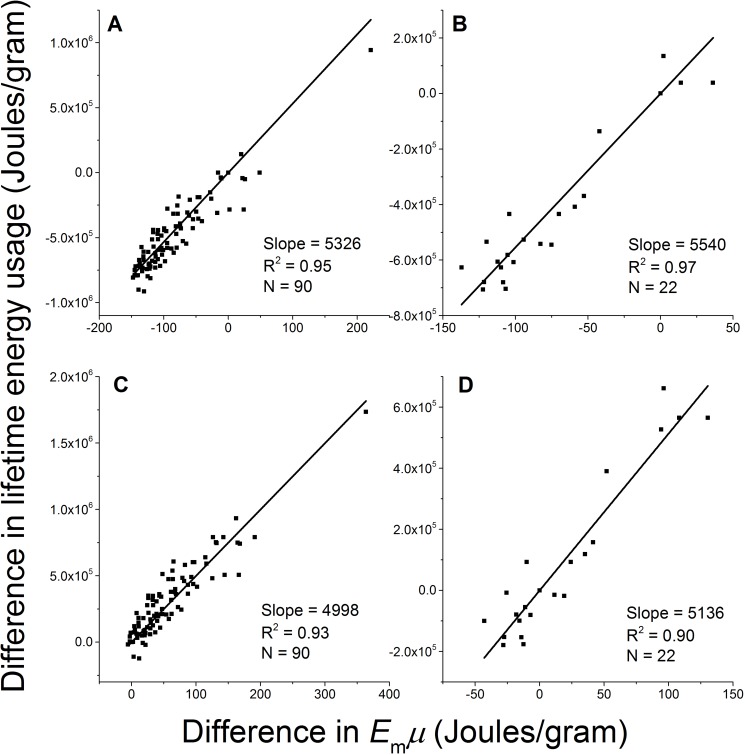
The difference in mass-specific lifetime energy usage is proportional to the difference in birth/adult mass ratio between breeds Four examples of fitting Eq. 2 with empirical data and fixing intercept at zero using (**A**) male Italian greyhound (M=4000 gram), (**B**) female Colon de Tulear (M=6250 gram), (**C**) male St. Bernard (M=70500 gram), and (**D**) female Chinook (M=25000 gram) as references.

We now test the prediction by Eq.2: the difference in lifetime energy usage (*ΔLE*) between breeds is proportional to the difference in birth/adult mass ratio (*Δμ*) multiplied by the energy for synthesizing one unit of biomass (*E*_m_) with a constant *b* = *ε*/(1−*ε*), i.e., *ΔLE* = *b×E*_m_*Δμ*. Within each gender, we took one breed as the reference (*j*), and calculated *ΔLE* = *LE_i_* − *LE_j_* and *E*_m_*Δμ* = *E*_m_(*μ_i_* − *μ_j_*) between other breeds (*i*'s) and this reference breed. We then linearly regressed Δ*LE* on *E*_m_*Δμ* in two ways, fixing the intercept at zero in accordance with Eq. 2 and letting it float to allow variation. We took all the breeds as the reference in turn, and obtained 90 male sets and 22 female sets of Δ*LE* versus *E*_m_*Δμ*, each containing 90 and 22 data points. Figure 4A-4D show four examples of the results using small breeds and large breeds as the references. The regression results of all the male and female sets are listed in Table 1. When intercept was fixed at zero as it is in Eq 2, the standard deviation of the fitted slopes among all the sets is small (coefficient of variation, SD/mean, of the slopes = ±15%). When the intercept is allowed to float, the fitted slope is the same regardless of which breed is used as the reference but the intercept varies. The fitted slope when intercept allowed to float is close to the mean value with a fixed intercept, and the fitted intercepts among all the datasets are normally distributed with the center at zero (−1< skewness <1). These results indicate that a constant slope with zero intercept is reasonable. The constant slope from the linear regression and the high R^2^ values strongly support our prediction that the mass-specific lifetime energy usage is proportional to the birth/adult mass ratio.

**Table 1:** Linear regression results of Eq. 2

	Regression method	Slope (Mean ± S.D.)	Intercept (Mean ± S.D.)	R^2^ value (Mean ± S.D.)	Fisher skewness
Male (90 sets)	Fixed intercept = 0	4623 ± 720	0	0.74 ± 0.15	−0.267
	Floating intercept	4572	−1.66×10^−11^±109713	0.849	0.00782
Female (22 sets)	Fixed intercept = 0	5164 ± 750	0	0.84 ± 0.07	−0.0545
	Floating intercept	5138	3.46×10^−11^± 86383	0.897	−0.619

Linear regression results of Eq. 2, *LE_i_* − *LE_j_* = *b×E*_m_(*μ_i_* − *μ_j_*) using data from male and female dogs, where *LE_i_* − *LE_j_* is the difference in mass-specific lifetime energy usage between the reference dog breed (*j*) and other breeds (*i*'s) in unit of energy/mass (joules/gram), and *E*_m_(*μ_i_* − *μ_j_*) is the difference in birth/adult mass ratio between other breeds (*i*'s) and the reference breed (*j*) multiplied by the energy required to synthesis one unit of biomass, *E*_m_, also in unit of energy/mass (joules/gram). Thus, the slope of the linear regression, *b*, is unitless. For male dogs, we took 90 breeds as the reference breed (*j*) in turn, and obtained 90 sets of regression, and for female dogs, we obtained 22 sets. This table shows the mean values and standard deviation of the slopes and intercepts of these regressions.

## DISCUSSION

We have presented a theoretical model based on the first principles in attempt to explain the negative correlation between lifespan and body size of dogs. The essence of the model lies in the energy tradeoff between somatic maintenance and biosynthesis. In recent years, increasing empirical evidence show such a tradeoff at the cellular/molecular level. For example, the growth-promoting pathways such as mTOR (mechanistic Target of Rapamycin) also drive aging [54, 55]. Another example is that high uncoupling protein expression in mitochondria slows down growth, and also reduces ROS production and cellular damage [56]. While such studies have helped to identify the mediators of the tradeoff at the molecular and cellular level, our theoretical model at the whole organismal level offers a collective framework that quantitatively analyzes the integrative and synergetic effects of these molecular pathways. Using generic principles, the model specifies the detailed energy budget underlying the tradeoff between growth and longevity, and makes quantitative predictions that are strongly supported by the empirical data.

One of the results of our model is that the scaling powers of birth/adult mass ratio of dogs plays an important role in the negative correlation between lifespan and adult size. Using data from 90 breeds of dogs, we estimated this scaling power to be around −0.55 (Figure 3). This is close to the previous finding, −0.44, which was obtained from a smaller sample size (N=8, R^2^ = 0.97) [7]. The strong negative scaling powers of the birth/adult mass ratio in dogs are sharply different than that of across mammalian species. Life history theory models have commonly assumed this ratio to be a constant for mammals interspecifically (e.g., [57]). Recently, using a large dataset Hamilton, Davidson [58] concluded that this ratio across placental mammalian species weakly scales with adult mass to a power of −0.07.

Our model assumes a threshold damage level *C* for death, i.e., *D*(*t* = *LS*) = *C*, “because of the redundancy in biological systems and the physiological tolerance of subthreshold losses in function” Sohal and Orr [52]. The cellular damage is not organ-specific. Fleming, Creevy [59] have shown that dogs of different breeds with different size die for different causes, such as gastrointestinal causes for large breeds and endocrine causes for small breeds. Although many of these causes can be attributed to damages in macromolecules [52], it is impossible that the thresholds of damage to cause function losses in different organs or death are exactly the same, as assumed in our theoretical model. If it varies in a range, i.e., *C*′ = *C* + *σ*, where σ is an organ-specific correction to the threshold, then we will have *D_i_*(*LS_i_*) = *D_j_*(*LS_j_*) + *Error*(*σ*), and our Eq. 2 will become Δ*LE* = *E*_m_*ε* / (1 − *ε*)Δ*μ* + *F*(*σ*), where *F*(*σ*) is a *σ*-dependent constant. Thus, if we plot *ΔLE* against *Δμ*, we will obtain a line with a slope of *E*_m_*ε*/(1−*ε*) and an intercept of *F*(*σ*). The linear regressions with floating intercept shown in Table 1 agree with this prediction, where the intercepts normally distribute around zero.

Kraus et al [13] recently found from mortality curves of 74 breeds of dogs that larger breeds have shorter lifespan because they have faster aging rate. Aging rate is mainly a statistical concept based on the mortality curves, and the physiological foundation of it remains unclear. It is possible that aging rate is linked to rate of damage accumulation, i.e., the faster the damage accumulates, the faster the aging is. The quantitative details of the relationship between aging rate and damage is unknown. However, our model predicts that larger breeds have faster damage accumulation rates (an example shown in Figure 2). So, if aging rate is indeed related to damage accumulation rate, then qualitatively our model makes a conclusion that agrees with what Kraus et al found.

The biosynthesis discussed in this paper only involves growth. Nonetheless, reproduction is another important process that requires a considerable amount of energy for biosynthesis, and therefore presumably also channels energy from health maintenance, as many researchers have suggested (e.g., see [60]). In this paper, we did not address the potential effects of reproduction on longevity for three reasons. First, the data on litter size of dogs are not available to us. Second, the data from male dogs agree with our model very well, whereas the biosynthetic requirement for male dogs’ reproduction is presumably minimal. Third, and more importantly, there is no quantitative understanding on how energetically costly mammalian reproduction is. Some researchers assume that reproduction simply diverts the biosynthetic effort from self-growth to offspring production, and modeled the energy cost of reproduction based on this assumption (e.g., [57]. However, energy content of bio-tissue is not equal to the energy required to synthesize the tissue [40], and the latter is the parameter *E*_m_ in our model. That is to say, even if one unit of biomass of the fetus has the same amount of combustion energy (energy content) as that of the mother, the amount of metabolic energy spent on synthesizing them may be different. For example, Hou, Bolt [33] have found that in some mammalian species, the energy cost for biosynthesis (*E*_m_) is about 4-fold cheaper for fetal development than that for post-natal growth. Thus, although reproduction has been shown to tradeoff with longevity in some species, such as fruit flies [61], a theoretical model based on the first principles and quantitative data analysis are yet to be developed.

Three important points of our results need clarification. First, the tradeoff between biosynthesis and maintenance is not imposed by the food supply, which is usually unlimited for domestic dogs. It is imposed by the fact that resting metabolic rate is roughly fixed for a given body size. The typical energy budget models (e.g., [40-42, 62]) partition the field metabolic rate (*B*) between the rates of energy for maintenance (*B*_maint_), the energy required for biosynthesizing new bio-tissues during growth (*B*_syn_), and the rate of energy spent on activities (*B*_act_), i.e., *B* = *B*_maint_ + *B*_syn_ + *B*_act_. The sum of the first two, *B*_maint_ and *B*_syn_, is the resting metabolic rate [40-42, 62]. For free-ranging animals, the ratio of field and resting metabolic rate is approximately a constant, so the energy cost for activities (*B*_act_ = *B* − *B*_rest_) is also a constant fraction of field metabolic rate *B* [3, 43, 53]. Thus, for a given body mass during growth, the energy available for both maintenance and biosynthesis only varies in a very narrow range, if there is no experimental manipulations. This narrow range is the reason for the tradeoff between *B*_maint_ and *B*_syn_. Food supply may be unlimited, but animals typically do not uptake more than the amount that is roughly determined by their body size, if there is no environmental or experimental stresses [40, 62, 63]. Nonetheless, many environmental stresses or experimental manipulations, such as cold exposure and forced-exercise, can change the total energy intake and energy partition. For example, heavy exercise increases animal's field metabolic rate. If food supply is unlimited, animals can simply increase the food intake to meet the increased demand imposed by the exercises [64]. In this case, the increase in field metabolic rate (*B*) comes from the increase in *B*_act_, but the resting metabolic rate, which is mainly determined by their body mass as *B*_o_*m^α^*, will not change, so that the tradeoff between maintenance and biosynthesis will keep the same. But, if animals are under food restriction, then long-term heavy exercises will suppress growth and reshuffle the energy budget. Depending on the degree of the exercises, the impacts on health maintenance may vary from negative, none, to positive (see detailed discussion in [19]). For domestic dogs discussed in this paper, some breeds may have higher mass-specific activity-induced energy cost than the others. But since the food supply is generally unlimited, the resting metabolic rate will not be affected by the exercise, and therefore the tradeoff between maintenance and growth will not be affected either, in general.

Second, there is only one free floating parameter in Eq.2—the species-specific protective efficiency *ε*, which expresses the ratio of damage repair and damage generation. Although *ε* cannot be directly measured, the fittings of data from more than 200 rodents and the theoretical estimate of protein oxidative damage and repair in mammals suggest that the value of *ε* is around 0.99 [18, 33]. From the fitted slopes *ε* / (1 − *ε*) in Figure 4, we estimate on average *ε* ≈ 0.999 for dogs, remarkably close to the previously estimated values. Our model assumes that *ε* is a constant for all the dog breeds. This assumption is supported by the following analysis of the sensitivity of the fitted slopes to *ε*. We denote the slope as *S* = *ε*/(1−*ε*). We take the derivative of *S* with respect to *ε*, and obtain (Δ*S* / *S*) = [1/(1 − *ε*)]×(Δ*ε* / *ε*), where Δ*S*/*S* and Δ*ε*/*ε* are the percentage changes in *S* and *ε* respectively. This equation shows that the percentage change in the slope *S* is proportional to the percentage change in *ε* with a coefficient [1/(1−*ε*)]. Using the average value of *ε* estimated from Figure 4, *ε* ∼ 0.999, we have [1/(1−ε)]∼1000. I.e., Δ*S*/*S* ∼ 1000 Δ*ε*/*ε*. Thus, a one percentage change in *ε* (Δ*ε*/*ε* = 0.01) will cause a 10-fold change (1000%) in Δ*S*/*S*. Table 1 shows that the variation in the slope *S* is only about 15% (SDT/mean), so we conclude that *ε* can be considered a constant across species.

Third, and more importantly, we need to emphasize that the relationship between *LE* and *μ* predicted by Eq.2 is general, and it can be applied to three kinds of special cases. The special case investigated in this study is dog breeds with different birth masses and adult masses, and therefore different ratios. As an indicator for energy allocated to biosynthesis, a higher *μ* means more energy allocated to maintenance, and therefore a longer lifespan. In a previous study [18], we applied Eq. 2 to explain another special case—the lifespan extension by food restriction in rodents, in which each pair of *i* (food restricted) and *j* (*ad libitum* free fed controls) has the same birth mass (*m*_0_), but different adult masses due to different food supply levels. Thus, Eq. 2 becomes

$$\begin{array}{l} B_{0}M_{\mathrm{FR}}^{\alpha -1}LS_{\mathrm{FR}}-B_{0}M_{\mathrm{AL}}^{\alpha -1}LS_{\mathrm{AL}}\\ =\frac{\varepsilon E_{m}}{1-\varepsilon }(\frac{m_{0}}{M_{\mathrm{FR}}}-\frac{m_{0}}{M_{\mathrm{AL}}})\\ =\frac{\varepsilon E_{m}}{1-\varepsilon }\frac{m_{0}}{M_{\mathrm{AL}}}(\frac{m_{\mathrm{AL}}}{M_{\mathrm{FR}}}-1) \end{array}$$

where subscripts “FR” and “AL” stand for food restriction and *ad libitum*, respectively. Since the adult mass of the AL control is usually larger than that of food restricted animals, (*M*_AL_/*M*_FR_ – 1) can be considered the relative reduction in body mass caused by the food restriction treatment. Thus, this equation predicts that the difference in lifetime energy usage between the restricted and control animals (left-hand side of the equation) is proportional to body mass reduction with a species-specific constant $\frac{\varepsilon E_{m}}{1-\varepsilon }\frac{m_{0}}{M_{AL}}$. Data from more than 200 studies on rodents strongly support this prediction [18]. Finally, across mammalian species the birth mass and adult mass both vary, but their ratio stays roughly a constant, and the right-hand side of Eq. 2 reduces to zero, i.e., *μ_i_* − *μ_j_* = 0. In this case, Eq. 2 predicts that mass-specific lifetime energy usage is approximately a constant, and that is exactly what the rate of living theory suggests and what has been observed across mammalian species.

A previous life history model [57, 65] suggested that the positive scaling power of lifespan across mammalian species stems from maximizing the net reproductive rate of non-growing populations with respect to maturation age, taking consideration of external mortality before maturity. Our model, on the other hand, highlights the importance of the physiological basis of the lifespan scaling laws. For domestic dogs, the artificial selection perhaps targets body size (and growth rate) along with other traits, such as personality, instead of net reproductive rate. Through the energy tradeoff between biosynthetic cost and health maintenance revealed in this study, the variation in body size (and birth/adult mass ratio) leads to the variation in lifespan in dogs. Lifespan extension by food restriction, which is usually conducted within one generation, gives prominence to the physiological basis, especially the plasticity of growth, even more. Since both Charnov's life history model and our physiological model derive the interspecific lifespan scaling law, there must be a bridge and perhaps some common hidden assumptions that connect these models. It requires future research to reconcile these models and integrate the relevant physiological and life history traits, as well as the environmental factors, for a general unified theory of lifespan scaling laws.

## SUPPLEMENTARY MATERIAL








