# Supplementary material for: Birth mass is the key to understanding the negative correlation between lifespan and body size in dogs
Source: Aging (Albany NY). 2016 Dec 8;8(12):3209–21. doi: 10.18632/aging.101081 (PMC5270664; doi:10.18632/aging.101081)
Supplement: Supplementary file 1 [file aging-08-3209-s001.pdf]

SUPPLEMENTARY MATERIAL

Please browse the links in Full Text version of this manuscript to see Supplementary Tables.

Supplementary Table S1. The birth mass, adult mass, and lifespan of 90 breeds of male dogs.

Supplementary Table S2. The birth mass, adult mass, and lifespan of 90 breeds of female dogs.
