# Supplementary material for: Birth mass is the key to understanding the negative correlation between lifespan and body size in dogs
Source: Aging (Albany NY). 2016 Dec 8;8(12):3209–21. doi: 10.18632/aging.101081 (PMC5270664; doi:10.18632/aging.101081)
Supplement: Supplementary file 2 [file aging-08-3209-s002.docx]

**Table S1. The birth mass, adult mass, and lifespan of 90 breeds of male dogs**

| Breed | Birth Mass (gram) | reference | Adult Mass (gram) | reference | Life span (years) | reference |
| --- | --- | --- | --- | --- | --- | --- |
| Affenpinscher | 86 | (1) | 4876 | (2) | 11 | (3) |
| Airedale Terrier | 397 | (4) | 21050 | (5) | 11.5 | (6) |
| Alaskan Malamute | 482 | (7) | 39000 | (8) | 10.7 | (9) |
| Australian Cattle Dog | 300 | (10) | 20500 | (11) | 13.5 | (12) |
| Basset hound | 450 | (13) | 10886 | (14) | 14 | (15) |
| Bearded Collie | 298 | (16) | 22500 | (17) | 14.5 | (18) |
| Beauceron | 481 | (19) | 34250 | (20) | 12 | (21) |
| Bernese Mountain Dog | 520 | (22) | 44000 | (23) | 11.5 | (24) |
| Black Russian Terrier | 667 | (25) | 55000 | (26) | 11.5 | (27) |
| Bloodhound | 450 | (28) | 45345 | (29) | 11 | (30) |
| Border Collie | 372 | (31) | 17000 | (32) | 13.5 | (33) |
| Border Terrier | 184 | (34) | 7485 | (35) | 13.5 | (36) |
| Borzoi | 359 | (37) | 41000 | (38) | 11 | (39) |
| Bouvier des Flandres | 326 | (40) | 42000 | (41) | 11 | (42) |
| Boxer | 482 | (43) | 29500 | (44) | 12.5 | (45) |
| Briard | 348 | (46) | 34050 | (47) | 12 | (48) |
| Brittany | 283 | (49) | 17000 | (50) | 13.5 | (51) |
| Brussels Griffon | 170 | (52) | 4080 | (53) | 13 | (54) |
| Bull Terrier | 283 | (55) | 27690 | (56) | 12 | (57) |
| Bulldog | 340 | (58) | 24500 | (59) | 10 | (60) |
| Bullmastiff | 635 | (61) | 55000 | (62) | 10 | (63) |
| Cairn Terrier | 193 | (64) | 7000 | (65) | 13.5 | (66) |
| Canaan Dog | 418 | (67) | 20500 | (68) | 13.5 | (69) |
| Cane Corso | 465 | (70) | 47500 | (71) | 10.5 | (72) |
| Cardigan Welsh Corgi | 340 | (73) | 12500 | (74) | 13.5 | (75) |
| Cavalier King Charles Spaniel | 210 | (76) | 6345 | (77) | 11.5 | (78) |
| Chihuahua | 113 | (79) | 1405 | (80) | 17.5 | (81) |
| Chinese Crested | 208 | (82) | 4500 | (83) | 14 | (84) |
| Chinook | 376 | (85) | 32000 | (86) | 12.5 | (87) |
| Cocker Spaniel | 250 | (88) | 10500 | (89) | 13.5 | (90) |
| Collie | 482 | (91) | 30500 | (92) | 15 | (93) |
| Coton de Tulear | 214 | (94) | 6250 | (95) | 15 | (96) |
| Curly-Coated Retriever | 358 | (97) | 37421 | (98) | 10.5 | (99) |
| Dachshund | 227 | (100) | 10880 | (101) | 13.5 | (102) |
| Dalmatian | 404 | (103) | 23360 | (104) | 14.5 | (105) |
| Doberman Pinscher | 312 | (106) | 35000 | (107) | 11.5 | (108) |
| English Cocker Spaniel | 313 | (109) | 14500 | (110) | 13.5 | (111) |
| English Setter | 482 | (112) | 30500 | (113) | 13 | (114) |
| English Springer Spaniel | 300 | (115) | 22500 | (116) | 12 | (117) |
| English Toy Spaniel（King Charles Spaniel） | 184 | (118) | 4989 | (119) | 11 | (120) |
| Field Spaniel | 283 | (121) | 19250 | (122) | 12.5 | (123) |
| Finnish Lapphund | 363 | (124) | 20640 | (125) | 11.5 | (126) |
| Flat-Coated Retriever | 284 | (127) | 29500 | (128) | 13 | (129) |
| French Bulldog | 255 | (130) | 9979 | (131) | 12.5 | (132) |
| German Pinscher | 265 | (133) | 15875 | (134) | 13 | (135) |
| German Shepherd Dog | 567 | (136) | 38555 | (137) | 12 | (138) |
| German Shorthaired Pointer | 500 | (139) | 28500 | (140) | 13.5 | (141) |
| Golden Retriever | 411 | (142) | 31750 | (143) | 11 | (144) |
| Gordon Setter | 315 | (145) | 30617 | (146) | 11 | (147) |
| Great Dane | 625 | (148) | 72000 | (149) | 8 | (150) |
| Great Pyrenees | 624 | (151) | 45000 | (152) | 11 | (153) |
| Greater Swiss Mountain Dog | 510 | (154) | 60000 | (155) | 10.5 | (156) |
| Greyhound | 550 | (157) | 32500 | (158) | 11 | (159) |
| Havanese | 156 | (160) | 4530 | (161) | 13.5 | (162) |
| Icelandic Sheepdog | 293 | (163) | 11500 | (164) | 12 | (165) |
| Irish Red and White Setter | 410 | (166) | 29500 | (167) | 12 | (168) |
| Irish Terrier | 280 | (169) | 11500 | (170) | 13.5 | (171) |
| Irish Wolfhound | 568 | (172) | 54500 | (173) | 7 | (174) |
| Italian Greyhound | 146 | (175) | 4000 | (176) | 13.5 | (177) |
| Keeshond | 255 | (178) | 19275 | (179) | 13.5 | (180) |
| Kuvasz | 547 | (181) | 48500 | (182) | 11 | (183) |
| Labrador retriever | 450 | (184) | 29800 | (185) | 12.6 | (186) |
| Leonberger | 510 | (187) | 68000 | (188) | 11 | (189) |
| Maltese | 142 | (190) | 3500 | (191) | 14.5 | (192) |
| English mastiff | 567 | (193) | 81645 | (194) | 11 | (195) |
| Miniature Pinscher | 184 | (196) | 4500 | (197) | 13.5 | (198) |
| Neapolitan Mastiff | 900 | (199) | 72574 | (200) | 10.5 | (201) |
| Norwegian Buhund | 345 | (202) | 16000 | (203) | 13.5 | (204) |
| Nova Scotia Duck Tolling Retriever | 470 | (205) | 20000 | (206) | 12 | (207) |
| Old English Sheepdog | 330 | (208) | 36275 | (209) | 11 | (210) |
| Otterhound | 631 | (211) | 41000 | (212) | 11 | (213) |
| Papillon | 150 | (214) | 4500 | (215) | 14 | (216) |
| Parson Russell Terrier | 190 | (217) | 7000 | (218) | 13.5 | (219) |
| Pembroke Welsh Corgi | 340 | (220) | 12000 | (221) | 13 | (222) |
| Portuguese Water Dog | 284 | (223) | 22000 | (224) | 13 | (225) |
| Pug | 227 | (226) | 7255 | (227) | 13.5 | (228) |
| Rat Terrier | 227 | (229) | 10750 | (230) | 15.5 | (231) |
| Redbone Coonhound | 439 | (232) | 27500 | (233) | 11.5 | (234) |
| Rhodesian Ridgeback | 440 | (235) | 36300 | (236) | 11 | (237) |
| Schipperke | 140 | (238) | 6750 | (239) | 12.5 | (240) |
| Shih Tzu | 170 | (241) | 5500 | (242) | 13 | (243) |
| Siberian Husky | 527 | (244) | 23500 | (245) | 13 | (246) |
| Smooth Fox Terrier | 213 | (247) | 8000 | (248) | 12.5 | (249) |
| Soft Coated Wheaten Terrier | 265 | (250) | 18000 | (251) | 13.5 | (252) |
| Spinone Italiano | 650 | (253) | 33112 | (254) | 12.5 | (255) |
| St. Bernard | 566 | (256) | 70500 | (257) | 11 | (258) |
| Staffordshire Bull Terrier | 284 | (259) | 14000 | (260) | 13 | (261) |
| Tibetan Mastiff | 510 | (262) | 67900 | (263) | 12 | (264) |
| Tibetan Terrier | 213 | (265) | 10900 | (266) | 13.5 | (267) |
| Vizsla | 482 | (268) | 23500 | (269) | 12 | (270) |
|  |  |  |  |  |  |  |
