# Supplementary material for: Birth mass is the key to understanding the negative correlation between lifespan and body size in dogs
Source: Aging (Albany NY). 2016 Dec 8;8(12):3209–21. doi: 10.18632/aging.101081 (PMC5270664; doi:10.18632/aging.101081)
Supplement: Supplementary file 3 [file aging-08-3209-s003.docx]

**Table S2. The birth mass, adult mass, and lifespan of 90 breeds of female dogs**

| Breed | Birth Mass (gram) | reference | Adult Mass (gram) | reference | Life span (years) | reference |
| --- | --- | --- | --- | --- | --- | --- |
| Airedale Terrier | 397 | (1) | 21050 | (2) | 11.5 | (3) |
| Australian Cattle Dog | 267 | (4) | 20500 | (5) | 13.5 | (6) |
| Beauceron | 466 | (7) | 34250 | (8) | 12 | (9) |
| Black Russian Terrier | 579 | (10) | 47500 | (11) | 11.5 | (12) |
| Border Collie | 362 | (13) | 15500 | (14) | 13.5 | (15) |
| Border Terrier | 170 | (16) | 6685 | (17) | 13.5 | (18) |
| Borzoi | 392 | (19) | 41000 | (20) | 11 | (21) |
| Briard | 389 | (22) | 34050 | (23) | 12 | (24) |
| Canaan Dog | 408 | (25) | 20500 | (26) | 13.5 | (27) |
| Cane Corso | 400 | (28) | 42500 | (29) | 10.5 | (30) |
| Chinook | 376 | (31) | 25000 | (32) | 12.5 | (33) |
| Coton de Tulear | 212 | (34) | 6250 | (35) | 15 | (36) |
| Finnish Lapphund | 358 | (37) | 20640 | (38) | 11.5 | (39) |
| Flat-Coated Retriever | 292 | (40) | 29500 | (41) | 13 | (42) |
| German Shepherd Dog | 248 | (43) | 38555 | (44) | 12 | (45) |
| Greyhound | 345 | (46) | 29000 | (47) | 11 | (48) |
| Icelandic Sheepdog | 276 | (49) | 12500 | (50) | 12 | (51) |
| Irish Red and White Setter | 377 | (52) | 29500 | (53) | 12 | (54) |
| Italian Greyhound | 147 | (55) | 4000 | (56) | 13.5 | (57) |
| Kuvasz | 608 | (58) | 48500 | (59) | 11 | (60) |
| Miniature Pinscher | 164 | (61) | 4000 | (62) | 13.5 | (63) |
| Papillon | 120 | (64) | 3500 | (65) | 14 | (66) |
