# Supplementary material for: Birth mass is the key to understanding the negative correlation between lifespan and body size in dogs
Source: Aging (Albany NY). 2016 Dec 8;8(12):3209–21. doi: 10.18632/aging.101081 (PMC5270664; doi:10.18632/aging.101081)
Supplement: Supplementary file 4 [file aging-08-3209-s004.docx]

**REFERENCES**

|  | <http://www.imrun.net/index.php?id=1072&view=view> |
| --- | --- |
|  | <http://www.ukcdogs.com/Web.nsf/Breeds/Affenpinscher04152007>  https://en.wikipedia.org/wiki/Affenpinscher |
|  | <http://www.dogbreedinfo.com/affenpinscher.htm> |
|  | http://www.woodcrestkennel.com/nitro-x-jada-2011/ |
|  | http://www.dogsindepth.com/terrier_dog_breeds/airedale.html |
|  | http://dogtime.com/dog-breeds/airedale-terrier |
|  | https://books.google.com/books?id=r3jBBAAAQBAJ&pg=PA12&lpg=PA12&dq=Alaskan+Malamute+birth+weight&source=bl&ots=j0NIrYMzQ8&sig=1LDTZR10-Vmt836pm2F8VLIcGEg&hl=zh-CN&sa=X&ei=ArhbVfexGMKSsAWo2oCADQ&ved=0CCMQ6AEwATge#v=onepage&q=Alaskan%20Malamute%20birth%20weight&f=false |
|  | http://en.wikipedia.org/wiki/Alaskan_Malamute |
|  | https://books.google.com/books?id=r3jBBAAAQBAJ&pg=PA12&lpg=PA12&dq=Alaskan+Malamute+birth+weight&source=bl&ots=j0NIrYMzQ8&sig=1LDTZR10-Vmt836pm2F8VLIcGEg&hl=zh-CN&sa=X&ei=ArhbVfexGMKSsAWo2oCADQ&ved=0CCMQ6AEwATge#v=onepage&q=Alaskan%20Malamute%20birth%20weight&f=false |
|  | http://horsemansbuddies.de/c-wurf%20-%20englisch.htm |
|  | http://www.dogbreedinfo.com/australiancattledog.htm |
|  | http://horsemansbuddies.de/c-wurf%20-%20englisch.htm |
|  | www.shecarabassets.com/exercise.html |
|  | http://www.animalplanet.com/breed-selector/dog-breeds/hound/basenji.html |
|  | http://dogtime.com/dog-breeds/basenji |
|  | http://skyebucksbeardedcollies.webs.com/apps/blog/entries?fb_sig_network=fw&fw_sig=a4f5644f49ab37c0d6c43d982d0627b1&fw_sig_access_token=13e0a3e7a4b8089c6b0027040ca238c704a109b6&fw_sig_api_key=ynwfbf8k2f7dn9jssqvcguxv&fw_sig_is_admin=0&fw_sig_locale=en-US&fw_sig_partner=webs&fw_sig_permission_level=0&fw_sig_permissions=none&fw_sig_premium=0&fw_sig_session_key=871d0e7e1dc9220522c3d17e3045f776dd41825503ff81dab83fa055bf8e336c-62412980&fw_sig_site=62412980&fw_sig_social=1&fw_sig_tier=0&fw_sig_time=1450971728134&fw_sig_url=http%3A%2F%2Fskyebucksbeardedcollies.webs.com%2F&page=2 |
|  | http://www.dogbreedinfo.com/beardedcollie.htm |
|  | http://pets.thenest.com/growth-weight-border-collies-9435.html |
|  | http://beauceron.zodasu.cz/en/odchovy.html |
|  | http://www.dogsindepth.com/herding_dog_breeds/beauceron.html |
|  | http://beauceron.zodasu.cz/en/odchovy.html |
|  | http://www.wccberners.com/previous-litters/litter-d-2/ |
|  | http://www.dogbreedinfo.com/bernesemountain.htm |
|  | http://www.bernese.biz/article11.htm https://books.google.com/books?id=F23pM3CcnMAC&pg=PA12&lpg=PA12&dq=Bernese+Mountain+Dog+birth+weight&source=bl&ots=mfB-ZghRP7&sig=7J_Y2azt63xtM8GALCzaal2eGXg&hl=zh-CN&sa=X&ei=uFSDVeW6MoP2sAWqhYSoBg&ved=0CEsQ6AEwBg#v=onepage&q=Bernese%20Mountain%20Dog%20birth%20weight&f=false |
|  | http://www.schwarzer-terrier.info/AWurfengl/birth1804.html |
|  | http://en.wikipedia.org/wiki/Black_Russian_Terrier |
|  | http://www.schwarzer-terrier.info/AWurfengl/birth1804.html |
|  | http://www.compassbreed.com/characteristics-of-the-bloodhound/ |
|  | http://www.dogsindepth.com/hound_dog_breeds/bloodhound.html |
|  | http://www.flessnerkennels.com/harveyslideshow.html https://books.google.com/books?id=itgJBgAAQBAJ&pg=PA19&lpg=PA19&dq=Bloodhound+birth+weight&source=bl&ots=PlghZjDSK8&sig=RFhG_bMGKE_cwwUqmFeGqLVjzXc&hl=zh-CN&sa=X&ei=E1KDVfeuEozpsAWl_YHQBQ&ved=0CGgQ6AEwCQ#v=onepage&q=Bloodhound%20birth%20weight&f=false |
|  | http://flame-of-phoenix.hu/eng/a_diary.htm |
|  | http://www.dogbreedinfo.com/bordercollie.htm |
|  | http://flame-of-phoenix.hu/eng/a_diary.htm |
|  | <http://rushwindlabs.com/border-terrier-puppies-2012> |
|  | <http://www.dogsindepth.com/terrier_dog_breeds/border_terrier.html> |
|  | <http://rushwindlabs.com/border-terrier-puppies-2012> |
|  | <http://kawailoaborzoi.blogspot.com/2012/02/its-official.html> |
|  | <http://www.dogbreedinfo.com/borzoi.htm> |
|  | <http://kawailoaborzoi.blogspot.com/2012/02/its-official.html> |
|  | <http://dutchbouviers.com/aboutus.htm> |
|  | <http://www.dogbreedinfo.com/bouvierdesflandres.htm> |
|  | <http://dutchbouviers.com/aboutus.htm> |
|  | <http://en.allexperts.com/q/Boxers-3345/2009/10/Boxer-Birth-Weight.htm> <https://boxerworld.com/forums/threads/average-birth-weight.12154/> |
|  | <http://www.dogbreedinfo.com/boxer.htm> |
|  | <http://en.allexperts.com/q/Boxers-3345/2009/10/Boxer-Birth-Weight.htm> <https://boxerworld.com/forums/threads/average-birth-weight.12154/> |
|  | <http://www.briardsauxpattesvelues.de/litters_a_en.html> |
|  | <http://www.dogsindepth.com/herding_dog_breeds/briard.html> |
|  | <http://www.briardsauxpattesvelues.de/litters_a_en.html>  <http://www.briardsauxpattesvelues.de/litters_a_en.html> |
|  | http://gundogforum.com/forum/viewtopic.php?f=88&t=23009 |
|  | http://www.dogbreedinfo.com/brittany.htm |
|  | http://gundogforum.com/forum/viewtopic.php?f=88&t=23009 |
|  | http://www.petforums.co.uk/threads/toy-breed-with-very-large-litter.396967/page-7 |
|  | http://www.dogsindepth.com/toy_dog_breeds/brussels_griffon.html |
|  | <http://pageland.yamvine.com/29728/pets-animals/brussels-griffon-male-akc_22344537.html>  http://dogtime.com/dog-breeds/brussels-griffon |
|  | https://books.google.com/books?id=615DAAAAYAAJ&pg=PA319&lpg=PA319&dq=bull+terrier+%22birth+weight%22&source=bl&ots=RELsV_9Ewx&sig=knEGbWiCnavx4BOUzA9kBcSRejY&hl=en&sa=X&ved=0CCQQ6AEwAWoVChMI4JSAwOr5xwIVhRWSCh3WWwtj#v=onepage&q=bull%20terrier%20%22birth%20weight%22&f=false |
|  | http://www.dogsindepth.com/terrier_dog_breeds/bull_terrier.html |
|  | http://www.andosstaffordshirebullterrier.yolasite.com/puppies-available.php https://books.google.com/books?id=615DAAAAYAAJ&pg=PA319&lpg=PA319&dq=Bull+Terrier+birth+weight&source=bl&ots=REKuY08Fyv&sig=z2_KktdXmhg94xiwwknFST430IA&hl=zh-CN&sa=X&ei=zEWDVa7HKMG-sAXA94JY&ved=0CCUQ6AEwATgU#v=onepage&q=Bull%20Terrier%20birth%20weight&f=false |
|  | http://pets.thenest.com/weight-progress-bulldog-puppies-4268.html |
|  | https://www.akc.org/breeds/bulldog/breed_standard.cfm |
|  | http://www.dogsindepth.com/nonsporting_dog_breeds/bulldog.html |
|  | http://lagratitudebullmastiffs.tumblr.com/post/57070755554/average-weights-of-my-bullmastiffs-female-male |
|  | http://www.dogbreedinfo.com/bullmastiff.htm |
|  | http://www.guardmansbullmastiffs.com/lu-lu-x-lucas-litter /http://lagratitudebullmastiffs.tumblr.com/post/57070755554/average-weights-of-my-bullmastiffs-female-male <http://www.hibullmoose.org/newpup.htm>  http://www.pet-owners.co.uk/Pet-Guides/Dogs/Dog-Breeds/Bullmastiff.aspx |
|  | https://www.youtube.com/watch?v=zoF14Y10Kg0 |
|  | http://www.dogbreedinfo.com/cairnterrier.htm |
|  | <https://www.youtube.com/watch?v=zoF14Y10Kg0>  http://dogs.petbreeds.com/l/42/Cairn-Terrier |
|  | http://kelev-storytelling.webnode.cz/new-canaan-dog-puppies/a-litter-of-canaan-dogs/ |
|  | http://www.dogbreedinfo.com/canaan.htm |
|  | http://kelev-storytelling.webnode.cz/new-canaan-dog-puppies/a-litter-of-canaan-dogs/ |
|  | http://www.qando.net/tag/cane-corso/ |
|  | http://www.dogbreedinfo.com/canecorsoitaliano.htm |
|  | http://www.qando.net/tag/cane-corso/ http://www.westcoastcanecorsos.com/available.html |
|  | https://books.google.com/books?id=rAVUBQAAQBAJ&pg=PT14&lpg=PT14&dq=Welsh+Corgi+%E2%80%9Cbirth+weight%E2%80%9D&source=bl&ots=bRQ-O2Y_OD&sig=pbnW7AWs5ngBlxQw7Mx6UxWew3E&hl=en&sa=X&ved=0CDMQ6AEwA2oVChMImdH-t-H5xwIVBl6SCh0ljw75#v=onepage&q=Welsh%20Corgi%20%E2%80%9Cbirth%20weight%E2%80%9D&f=false |
|  | http://www.dogbreedinfo.com/cardigancorgi.htm |
|  | http://www.k9web.com/dog-faqs/breeds/pembrokes.html https://books.google.com/books?id=rAVUBQAAQBAJ&pg=PT14&lpg=PT14&dq=Cardigan+Welsh+Corgi+birth+weight&source=bl&ots=bRP1R4YZNG&sig=ccwjm_WmVwRCL_Ic27npUZXVfxg&hl=zh-CN&sa=X&ei=Uj-DVY3xD8bYtQXx-IPwBg&ved=0CE4Q6AEwBjgK#v=onepage&q=Cardigan%20Welsh%20Corgi%20birth%20weight&f=false |
|  | http://www.goldenleon.cz/Puppies-Cavalier-King-Charles-spaniel-F.html |
|  | http://www.dogsindepth.com/toy_dog_breeds/cavalier_king_charles_spaniel.html |
|  | http://www.justanswer.com/pet-dog/0uxdh-litter-five-cavalier-king-charles-spaniels.html / http://forum.champdogs.co.uk/topic_show.pl?tid=58417 |
|  | http://teacupchihuahuas.info/chihuahua-weight-chart/ |
|  | http://www.dogsindepth.com/toy_dog_breeds/chihuahua.html |
|  | http://www.dogbreedinfo.com/chihuahua.htm |
|  | <http://www.hoobly.com/Yrtrk/introducing-c-litter.htm>  http://www.pet-classifieds.com/pet13787590.htm |
|  | http://www.dogbreedinfo.com/chinesecrested.htm |
|  | <http://www.hoobly.com/Yrtrk/introducing-c-litter.htm>  <http://dogs.petbreeds.com/l/48/Chinese-Crested>  http://www.pet-classifieds.com/pet13787590.htm |
|  | http://www.skylandchinooks.com/week%208.htm |
|  | http://www.dogbreedinfo.com/chinook.htm |
|  | http://www.skylandchinooks.com/week%208.htm |
|  | http://www.powerscourt-cockers.co.uk/advice_pages/cocker_spaniel_weight.htm |
|  | http://www.dogbreedinfo.com/americancocker.htm |
|  | http://www.powerscourt-cockers.co.uk/advice_pages/cocker_spaniel_weight.htm |
|  | http://www.chelsea-collies.com/newborn.html |
|  | http://www.dogbreedinfo.com/collie.htm |
|  | http://www.chelsea-collies.com/newborn.html |
|  | http://alikacoton.com/Chris.07/Chris.Litter.4.07_page1.html |
|  | http://www.dogbreedinfo.com/cotondetulear.htm |
|  | http://alikacoton.com/Chris.07/Chris.Litter.4.07_page1.html |
|  | http://www.zbasisfory.com/page/12/?li=24&lang=en |
|  | http://dogtime.com/dog-breeds/curly-coated-retriever |
|  | http://www.zbasisfory.com/page/12/?li=24&lang=en |
|  | https://books.google.com/books?id=vmTgPakg8nUC&pg=PA145&lpg=PA145&dq=dachshund+%2522birth+weight%2522&source=bl&ots=faNDPfAMr5&sig=lW8oh9CgIYFAoMWiTWPocnzc9uM&hl=en&sa=X&ved=0CB0Q6AEwAGoVChMI0qPC6KT-xwIVQQaSCh2bCw7C%23v=onepage&q=dachshund%2520%2522birth%2520weight%2522&f=false |
|  | http://www.dogsindepth.com/hound_dog_breeds/dachshund.html |
|  | http://www.vetstreet.com/dogs/dachshund |
|  | http://www.obonyas-pride.nl/wb/pages/english/obonyas-pride.php?lang=EN |
|  | http://dogtime.com/dog-breeds/dalmatian |
|  | http://www.obonyas-pride.nl/wb/pages/english/obonyas-pride.php?lang=EN |
|  | http://www.ladpc.org/Puppies.html |
|  | http://www.dogbreedinfo.com/doberman.htm |
|  | http://dogtime.com/dog-breeds/doberman-pinscher |
|  | http://lindenmarshdogs.weebly.com/lotties-puppies.html |
|  | http://www.dogbreedinfo.com/englishcocker.htm |
|  | http://lindenmarshdogs.weebly.com/lotties-puppies.html |
|  | http://www.englishsetterassociation.co.uk/Health/management.pdf |
|  | http://www.dogbreedinfo.com/englishsetter.htm |
|  | http://dogtime.com/dog-breeds/english-setter |
|  | http://www.justanswer.com/dog-health/2ft54-typical-length-newborn-english-springer-spaniel.html |
|  | http://www.dogbreedinfo.com/englishspringerspaniel.htm |
|  | http://dogtime.com/dog-breeds/english-springer-spaniel |
|  | http://forum.champdogs.co.uk/topic_show.pl?tid=58417 |
|  | http://dogtime.com/dog-breeds/english-toy-spaniel |
|  | http://www.dogbreedinfo.com/englishtoyspaniel.htm |
|  | http://www.justanswer.com/dog-health/3fcl5-day-old-field-spaniels-puppy-birth-weight-10-oz-10-3.html |
|  | http://www.dogbreedinfo.com/fieldspaniel.htm |
|  | http://www.dogsindepth.com/sporting_dog_breeds/field_spaniel.html |
|  | http://infindigo.blogspot.com/2012/02/riemu-puppies.html |
|  | http://www.dogsindepth.com/spitz_dog_breeds/finnish_lapphund.html |
|  | http://infindigo.blogspot.com/2012/02/riemu-puppies.html |
|  | http://www.flashbackfcr.com/Statistics1.htm |
|  | http://www.dogbreedinfo.com/flatcoatedretriever.htm |
|  | http://www.dogsindepth.com/sporting_dog_breeds/flat_coated_retriever.html |
|  | http://www.seattlefrenchbulldogs.com/LacyPup12/index.html |
|  | http://dogtime.com/dog-breeds/french-bulldog |
|  | http://www.seattlefrenchbulldogs.com/LacyPup12/index.html |
|  | http://harmony-star.wz.cz/en/nabidka.htm |
|  | http://dogtime.com/dog-breeds/german-pinscher |
|  | http://harmony-star.wz.cz/en/nabidka.htm |
|  | <http://www.shepherdpuppies.com.au/german-shepherd-puppy-growth-chart/>  http://sequoyah-german-shepherds.com/gsd_growth.htm |
|  | http://dogtime.com/dog-breeds/german-shepherd-dog |
|  | http://www.shepherdpuppies.com.au/german-shepherd-puppy-growth-chart/ |
|  | http://germanshorthairedpointer.pro/litter-c-is-here/ |
|  | http://www.dogbreedinfo.com/germanshorthairedpointer.htm |
|  | http://germanshorthairedpointer.pro/litter-c-is-here/ |
|  | http://www.goldenretrieverforum.com/golden-retriever-health-anatomy-physiology-breed-standard/33463-average-golden-puppy-length-weight-birth.html |
|  | http://www.dogsindepth.com/sporting_dog_breeds/golden_retriever.html |
|  | http://www.dogbreedinfo.com/goldenretriever.htm |
|  | http://gladysrosegordons.weebly.com/puppies.html |
|  | http://www.animalplanet.com/breed-selector/dog-breeds/sporting/gordon-setter.html |
|  | http://dogtime.com/dog-breeds/gordon-setter |
|  | <http://www.chromadane.com/index.php/en/chromalinx/83-general-breed-info/103-height-weight-data-for-danes>  <http://www.soveryshiny.com/great-dane-growth-chart.html>  http://www.greatdane-dog-world.com/great-dane-growth-chart.html  Pomeranian.pdf |
|  | http://www.dogbreedinfo.com/greatdane.htm |
|  | https://en.wikipedia.org/wiki/Great_Dane |
|  | http://www.snowbear-greatpyrenees.com/SummerPuppies/sumpup1.html |
|  | http://www.dogbreedinfo.com/greatpyrenees.htm |
|  | http://www.snowbear-greatpyrenees.com/SummerPuppies/sumpup1.html |
|  | http://www.bmdinfo.org/Litterlife/week_one.php |
|  | http://www.dogbreedinfo.com/greaterswissmountain.htm |
|  | http://www.dogsindepth.com/working_dog_breeds/greater_swiss_mountain_dog.html |
|  | http://www.greyhound-data.com/knowledge.php?b=3&note=1052580 |
|  | https://www.dogbreedinfo.com/greyhound.htm |
|  | http://www.dogsindepth.com/hound_dog_breeds/greyhound.html |
|  | http://www.havanese.org/index.php/education/breeders-education/101-newborn-care |
|  | http://www.dogsindepth.com/toy_dog_breeds/havanese.html |
|  | http://dogtime.com/dog-breeds/havanese |
|  | http://www.stubbornelmranch.com/icelandicsheepdogpuppies.html |
|  | http://www.dogbreedinfo.com/icelandicsheepdog.htm |
|  | http://www.stubbornelmranch.com/icelandicsheepdogpuppies.html |
|  | http://www.gilliegraesetters.co.uk/Cara-s-Litter-20-08-11.html |
|  | http://www.dogbreedinfo.com/irishredwhitesetter.htm |
|  | http://dogtime.com/dog-breeds/irish-red-and-white-setter |
|  | http://www.irish-terrier.de/en/zucht_entwicklung.html |
|  | https://www.dogbreedinfo.com/irishterrier.htm |
|  | http://www.irish-terrier.de/en/zucht_entwicklung.html |
|  | http://www.carroyirishwolfhounds.net/Puppies.html |
|  | http://www.dogbreedinfo.com/irishwolfhound.htm |
|  | http://dogtime.com/dog-breeds/irish-wolfhound |
|  | http://www.texasitaliangreyhounds.com/category/puppies-for-sale/ |
|  | http://www.dogbreedinfo.com/italiangreyhound.htm |
|  | http://www.texasitaliangreyhounds.com/category/puppies-for-sale/ |
|  | http://trumpetkees.phanfare.com/6325053 |
|  | http://www.dogsindepth.com/nonsporting_dog_breeds/keeshond.html |
|  | http://www.dogbreedinfo.com/keeshond.htm |
|  | http://www.ecarpadi.se/en/e-litter/ |
|  | http://www.dogbreedinfo.com/kuvasz.htm |
|  | http://dogtime.com/dog-breeds/kuvasz |
|  | http://labradornet.com/labweight.html |
|  | Speakman et al 2003 Age-related changes in the metabolism and body composition of three dog breeds and their relationship to life expectancy, Aging cell 2: 265-275 |
|  | Speakman et al 2003 Age-related changes in the metabolism and body composition of three dog breeds and their relationship to life expectancy, Aging cell 2: 265-275 |
|  | http://www.lionsfromdes.be/en/leonberger-puppies-%E2%80%9D-happyghostjes%E2%80%9D-n5-day-28 |
|  | http://www.dogbreedinfo.com/leonberger.htm |
|  | http://www.dogsindepth.com/molossoid_dog_breeds/leonberger.html |
|  | http://www.maltesemaniac.com/puppy-weight-chart.html |
|  | https://www.dogbreedinfo.com/maltese.htm |
|  | http://www.dogsindepth.com/toy_dog_breeds/maltese.html |
|  | https://answers.yahoo.com/question/index?qid=20150216062731AA3LcL1 http://www.mastiff-forum.com/showthread.php/24218-Mack-s-growth-chart.-He-s-a-big-guy!/page2 |
|  | http://www.dogsindepth.com/working_dog_breeds/mastiff.html |
|  | http://www.dogbreedinfo.com/mastiff.htm |
|  | http://www.dogster.com/dogs/977945 |
|  | http://www.dogbreedinfo.com/miniaturepinscher.htm |
|  | http://www.dogsindepth.com/toy_dog_breeds/miniature_pinscher.html |
|  | https://www.dogzonline.com.au/breeds/profile.asp?dog=57892 |
|  | http://dogtime.com/dog-breeds/neapolitan-mastiff |
|  | http://www.dogsindepth.com/working_dog_breeds/neapolitan_mastiff.html |
|  | http://buhund.eu/kennel/breed/g-litter/g.htm |
|  | http://www.dogbreedinfo.com/norwegianbuhund.htm |
|  | http://www.dogsindepth.com/working_dog_breeds/norwegian_buhund.html |
|  | http://www.redraisins.de/en/kennel/b-litter.php |
|  | http://www.dogbreedinfo.com/novascotiaducktolling.htm |
|  | http://dogtime.com/dog-breeds/nova-scotia-duck-tolling-retriever |
|  | http://www.bobtail.ru/text/oes/puppy_e.htm |
|  | http://www.dogsindepth.com/herding_dog_breeds/old_english_sheepdog.html |
|  | http://www.dogbreedinfo.com/oldenglishsheepdog.htm |
|  | http://www.riversprucehounds.com/2009%20Otterhound%20Puppy%20Weight%20Chart.html |
|  | http://www.dogbreedinfo.com/otterhound.htm |
|  | http://www.riversprucehounds.com/2009%20Otterhound%20Puppy%20Weight%20Chart.html |
|  | http://www.pinefields.eu/emiot_c.html |
|  | http://www.dogbreedinfo.com/papillon.htm |
|  | http://www.dogsindepth.com/toy_dog_breeds/papillon.html |
|  | http://www.mayabor.com/index.php/en/puppies |
|  | http://www.dogbreedinfo.com/parsonrussellterrier.htm |
|  | http://www.dogsindepth.com/terrier_dog_breeds/parson_russell_terrier.html |
|  | http://www.k9web.com/dog-faqs/breeds/pembrokes.html |
|  | http://www.dogbreedinfo.com/pembrokecorgi.htm |
|  | http://www.dogsindepth.com/herding_dog_breeds/pembroke_welsh_corgi.html |
|  | http://dogs.thehupps.com/puppies/violet/ |
|  | http://www.dogbreedinfo.com/portuguesewaterdog.htm |
|  | http://www.dogsindepth.com/working_dog_breeds/portuguese_water_dog.html |
|  | https://answers.yahoo.com/question/index?qid=20120608020754AANpKRn |
|  | http://www.dogsindepth.com/toy_dog_breeds/pug.html |
|  | http://dogtime.com/dog-breeds/pug |
|  | https://answers.yahoo.com/question/index?qid=20100721162038AAeJO84 |
|  | http://www.dogbreedinfo.com/ratterrier.htm |
|  | http://dogtime.com/dog-breeds/rat-terrier |
|  | http://trredbones.tripod.com/welcome/id220.html |
|  | http://www.dogbreedinfo.com/redbonecoonhound.htm |
|  | http://www.dogsindepth.com/hound_dog_breeds/redbone_coonhound.html |
|  | http://www.kaweria.com/english/diary_a_litter.php |
|  | http://www.dogsindepth.com/hound_dog_breeds/rhodesian_ridgeback.html |
|  | http://www.dogbreedinfo.com/rhodesianridgeback.htm |
|  | http://www.k9puppydogs.com/dogbreeds/schipperke-dog |
|  | http://www.dogbreedinfo.com/schipperke.htm |
|  | http://www.dogsindepth.com/nonsporting_dog_breeds/schipperke.html |
|  | http://www.shihtzuonly.com/Micky-Furby.html |
|  | http://www.dogbreedinfo.com/shihtzu.htm |
|  | http://dogtime.com/dog-breeds/shih-tzu |
|  | http://resapense.wix.com/husky-beautiful#!2012-puppies/c1v5i |
|  | http://www.dogbreedinfo.com/siberianhusky.htm |
|  | http://www.dogsindepth.com/working_dog_breeds/siberian_husky.html |
|  | http://www.afterallwirefoxterriers.com/18_Days_Old.htm |
|  | http://www.dogbreedinfo.com/smoothfoxterrier.htm |
|  | http://dogtime.com/dog-breeds/fox-terrier |
|  | Soft Coated Wheaten Terrier.pdf |
|  | http://www.dogbreedinfo.com/softcoatedwheatenterrier.htm |
|  | http://www.dogsindepth.com/terrier_dog_breeds/soft_coated_wheaten_terrier.html |
|  | http://www.itspinone.com/bramker04.htm |
|  | http://www.dogbreedinfo.com/spinone.htm |
|  | http://www.dogsindepth.com/sporting_dog_breeds/spinone_italiano.html |
|  | http://saintbernardclub.org/looking-for-a-saint-bernard/faq/ |
|  | http://www.dogbreedinfo.com/saintbernard.htm |
|  | http://www.dogsindepth.com/working_dog_breeds/saint_bernard.html |
|  | http://www.jajca.co.uk/testimonials_Details.asp |
|  | http://www.dogbreedinfo.com/staffordshirebullterrier.htm |
|  | http://dogtime.com/dog-breeds/staffordshire-bull-terrier |
|  | http://www.k9cabins.net/sanashoba/puppies.shtml |
|  | http://www.dogsindepth.com/molossoid_dog_breeds/tibetan_mastiff.html |
|  | http://dogtime.com/dog-breeds/tibetan-mastiff |
|  | http://juliehindle.wix.com/abelenus-tibetan-terriers#!__second-born |
|  | http://www.dogbreedinfo.com/tibetanterrier.htm |
|  | http://www.dogsindepth.com/nonsporting_dog_breeds/tibetan_terrier.html |
|  | http://hungarianvizsla.org.uk/grow.html |
|  | http://www.dogbreedinfo.com/vizsla.htm |
|  | http://dogtime.com/dog-breeds/vizsla |
|  | <http://www.woodcrestkennel.com/nitro-x-jada-2011/> |
|  | http://www.dogsindepth.com/terrier_dog_breeds/airedale.html |
|  | http://dogtime.com/dog-breeds/airedale-terrier |
|  | http://horsemansbuddies.de/c-wurf%20-%20englisch.htm |
|  | http://www.dogbreedinfo.com/australiancattledog.htm |
|  | http://horsemansbuddies.de/c-wurf%20-%20englisch.htm |
|  | http://beauceron.zodasu.cz/en/odchovy.html |
|  | http://www.dogsindepth.com/herding_dog_breeds/beauceron.html |
|  | http://beauceron.zodasu.cz/en/odchovy.html |
|  | http://www.schwarzer-terrier.info/AWurfengl/birth1804.html |
|  | http://en.wikipedia.org/wiki/Black_Russian_Terrier |
|  | http://www.schwarzer-terrier.info/AWurfengl/birth1804.html |
|  | http://flame-of-phoenix.hu/eng/a_diary.htm |
|  | http://www.dogbreedinfo.com/bordercollie.htm |
|  | http://flame-of-phoenix.hu/eng/a_diary.htm |
|  | http://rushwindlabs.com/border-terrier-puppies-2012 |
|  | http://www.dogsindepth.com/terrier_dog_breeds/border_terrier.html |
|  | http://rushwindlabs.com/border-terrier-puppies-2012 |
|  | http://kawailoaborzoi.blogspot.com/2012/02/its-official.html |
|  | http://www.dogbreedinfo.com/borzoi.htm |
|  | http://kawailoaborzoi.blogspot.com/2012/02/its-official.html |
|  | http://www.briardsauxpattesvelues.de/litters_a_en.html |
|  | http://www.dogsindepth.com/herding_dog_breeds/briard.html |
|  | http://www.briardsauxpattesvelues.de/litters_a_en.html |
|  | http://kelev-storytelling.webnode.cz/new-canaan-dog-puppies/a-litter-of-canaan-dogs/ |
|  | http://www.dogbreedinfo.com/canaan.htm |
|  | http://kelev-storytelling.webnode.cz/new-canaan-dog-puppies/a-litter-of-canaan-dogs/ |
|  | http://www.qando.net/tag/cane-corso/ |
|  | http://www.dogbreedinfo.com/canecorsoitaliano.htm |
|  | http://www.qando.net/tag/cane-corso/ http://www.westcoastcanecorsos.com/available.html |
|  | http://www.skylandchinooks.com/week%208.htm |
|  | http://www.dogbreedinfo.com/chinook.htm |
|  | http://www.skylandchinooks.com/week%208.htm |
|  | http://alikacoton.com/Chris.07/Chris.Litter.4.07_page1.html |
|  | http://www.dogbreedinfo.com/cotondetulear.htm |
|  | http://alikacoton.com/Chris.07/Chris.Litter.4.07_page1.html |
|  | http://infindigo.blogspot.com/2012/02/riemu-puppies.html |
|  | http://www.dogsindepth.com/spitz_dog_breeds/finnish_lapphund.html |
|  | http://infindigo.blogspot.com/2012/02/riemu-puppies.html |
|  | http://www.flashbackfcr.com/Statistics1.htm |
|  | http://www.dogbreedinfo.com/flatcoatedretriever.htm |
|  | http://www.dogsindepth.com/sporting_dog_breeds/flat_coated_retriever.html |
|  | http://www.shepherdpuppies.com.au/german-shepherd-puppy-growth-chart/  http://www.german-shepherd-lore.com/german-shepherd-growth-chart.html |
|  | http://dogtime.com/dog-breeds/german-shepherd-dog |
|  | http://www.shepherdpuppies.com.au/german-shepherd-puppy-growth-chart/  http://www.german-shepherd-lore.com/german-shepherd-growth-chart.html |
|  | http://www.greyhound-data.com/knowledge.php?b=2&note=723425 |
|  | https://www.dogbreedinfo.com/greyhound.htm |
|  | http://www.dogsindepth.com/hound_dog_breeds/greyhound.html |
|  | http://www.stubbornelmranch.com/icelandicsheepdogpuppies.html |
|  | https://www.vetarena.org/encyclopedia-dog-breeds/442/icelandic-sheepdog.html |
|  | http://www.stubbornelmranch.com/icelandicsheepdogpuppies.html |
|  | http://www.gilliegraesetters.co.uk/Cara-s-Litter-20-08-11.html |
|  | http://www.dogbreedinfo.com/irishredwhitesetter.htm |
|  | http://dogtime.com/dog-breeds/irish-red-and-white-setter |
|  | http://www.texasitaliangreyhounds.com/category/puppies-for-sale/ |
|  | http://www.dogbreedinfo.com/italiangreyhound.htm |
|  | http://www.texasitaliangreyhounds.com/category/puppies-for-sale/ |
|  | http://www.ecarpadi.se/en/e-litter/ |
|  | http://www.dogbreedinfo.com/kuvasz.htm |
|  | http://dogtime.com/dog-breeds/kuvasz |
|  | http://www.dogster.com/dogs/977946 |
|  | http://www.dogbreedinfo.com/miniaturepinscher.htm |
|  | http://www.dogsindepth.com/toy_dog_breeds/miniature_pinscher.html |
|  | http://www.pinefields.eu/emiot_c.html |
|  | http://www.dogbreedinfo.com/papillon.htm |
|  | http://www.dogsindepth.com/toy_dog_breeds/papillon.html |
